# Youth in a viral age: a collated auto‐ethnographic response by young people (dis)orientated in strange times

**DOI:** 10.1111/1469-8676.12815

**Published:** 2020-06-14

**Authors:** Rosalie Jones McVey, Izzy Clancy, Anna Curzon Price, Stella Rose Hall Dixon, Jiayu Qiu, Mingwei Song

**Affiliations:** ^1^ Department of Social Anthropology Cambridge University Cambridge CB2 3RF United Kingdom

Our experiences of COVID‐19 have caused us to reflect on youth as an orientation in time. Writing as mostly undergraduates from Cambridge University, before COVID‐19, we lived among expectations that young people’s time should be sociable, memorable, ambitious and self‐discovering. The modern world watches young people for evidence of ‘potential’ in a way that is both exciting and stifling. Young people’s actions are like timepieces: they act as measures for the emergence of new worlds. We might have been forging the future or allowing societal degradation: either way, our actions were timely.

Since COVID‐19, our potential futures have been threatened and suspended. ‘Young people are at less risk’, we are told. This is a fortunate, thankful, strangeness for us. Young people’s lives, in our normal times, are defined by risk. Now, we are in a new sort of accelerated standstill. We must hurry up and do nothing. Our inertness will buy time for our grandparents, our health service, our nations. We nervously wait for the pages to load. We are holding our breath for what is to come, and we are on hold. Our digital newsfeeds are clogged with a race against time, but we are not running. Ominous doom accompanies everyday boredom. This time will pass, we reassure one another, which is a strange reassurance.

With the world less urgently invested in our time, some of us are finding new freedoms. Some are enjoying nostalgia, playing games with our families and spending time with siblings. Screen‐time is no longer monitored with the same sense of ration. What was reclusive is now appropriate. Some of us find this is a time to experiment with alternative selves: new haircuts or hobbies. Though for some, time trapped in parental homes will stunt or scar our growth.

We are rethinking what we are to others, as a generation. We are finding new sorts of comparison between our youth and those of our war‐time relations. Our (presumed) technological prowess has become a wireless lifeline for others. Some of us apply ourselves to innovation: hackathons and other forms of technological creativity. Our families look to us to know how to use technology both to waste time and to make meaning. Some of us set up Facetime for those denied face‐to‐face time. We show them it will be OK, that digital relationships are real relationships – though in fact we are not always sure.

We liked it when sociality happened in hallways, by coincidence of schedule. We miss accidental, unedited intimacies and unfiltered proximities. Intercorporeal immediacy can’t be coded with emojis. We may all share this strange time of urgent suspension, but we are lonely, because we are not in responsive rhythm with one another’s bodies, in study sessions, dancing, drunkenness, sports or sex. We recognise, with hindsight, that it was the unpredictability of inadvertently bumping into one another, figuratively and literally, that made screenless interaction feel authentic.

In very different ways, both ‘youth’ and COVID‐19 could be investigated as devices that (dis)orientate people within time. Both encompass forms of unpredictability. By attending to the patchy way that COVID‐19 impacts on youth, we believe we have things to learn about the ethical, political and emotional states that are afforded by uncertain times.

